# Parameters Influencing Sulfur Speciation in Environmental Samples Using Sulfur K-Edge X-Ray Absorption Near-Edge Structure

**DOI:** 10.1155/2012/659858

**Published:** 2012-11-05

**Authors:** Siwatt Pongpiachan, Kanjana Thumanu, Charnwit Kositanont, Klaus Schwarzer, Jörg Prietzel, Phoosak Hirunyatrakul, Itthipon Kittikoon

**Affiliations:** ^1^NIDA Center for Research & Development of Disaster Prevention & Management, School of Social and Environmental Development, National Institute of Development Administration (NIDA), 118 Moo 3, Sereethai Road, Klong-Chan, Bangkapi, Bangkok 10240, Thailand; ^2^Synchrotron Light Research Institute (Public Organization), 111 University Avenue, P.O. Box 93, Nakhon Ratchasima 30000, Thailand; ^3^Inter-Department of Environmental Science, Faculty of Graduate Studies, Chulalongkorn University, Bangkok 10330, Thailand; ^4^Institute of Geosciences Sedimentology, Coastal and Continental Shelf Research, Otto-Hahn-Platz 1, 24118 Kiel, Germany; ^5^Lehrstuhl für Bodenkunde, Technische Universität München, Weihenstephan 85350 Freising, Germany; ^6^Bara Scientific Co., Ltd., 968 Rama 4 Silom Bangrak, Bangkok 10500, Thailand

## Abstract

This paper aims to enhance the credibility of applying the sulfur K-edge XANES spectroscopy as an innovative “fingerprint” for characterizing environmental samples. The sensitivities of sulfur K-edge XANES spectra of ten sulfur compound standards detected by two different detectors, namely, Lytle detector (LyD) and Germanium detector (GeD), were studied and compared. Further investigation on “self-absorption” effect revealed that the maximum sensitivities of sulfur K-edge XANES spectra were achieved when diluting sulfur compound standards with boron nitride (BN) at the mixing ratio of 0.1%. The “particle-size” effect on sulfur K-edge XANES spectrum sensitivities was examined by comparing signal-to-noise ratios of total suspended particles (TSP) and particulate matter of less than 10 millionths of a meter (PM_10_) collected at three major cities of Thailand. The analytical results have demonstrated that the signal-to-noise ratios of sulfur K-edge XANES spectra were positively correlated with sulfate content in aerosols and negatively connected with particle sizes. The combination of hierarchical cluster analysis (HCA) and principal component analysis (PCA) has proved that sulfur K-edge XANES spectrum can be used to characterize German terrestrial soils and Andaman coastal sediments. In addition, this study highlighted the capability of sulfur K-edge XANES spectra as an innovative “fingerprint” to distinguish tsunami backwash deposits (TBD) from typical marine sediments (TMS).

## 1. Introduction

During the past decades, considerable research has been intensively focused on the topic of sulfur speciation in the environment. It has become more evident that the plant uptake availability [1] and climate forcing capability [2, 3] of sulfur compounds depend not purely on their concentrations but, severely, on the chemical forms in which they exist in natural compartments. Furthermore, ammonium salts such as ammonium chloride (NH_4_Cl), ammonium nitrate (NH_4_NO_3_), ammonium sulfate ((NH_4_)_2_SO_4_), and bisulfate (NH_4_HSO_4_) are widely considered as acidic aerosols and may have been responsible for the acid rain in pristine area [4, 5]. Hence, information on the chemical state of sulfur in aerosol is important from the viewpoint of environmental science, in order to study the origins of the acid rain and the causes of climate change. 

Continuing developments in various atomic spectrochemical techniques enable researchers to extract some useful information related to the physicochemical properties of sulfur contents in atmospheric aerosols. For instance, the chemical speciation of sulfate aerosols collected weekly for a one-year period in Matsue, Shimane prefecture, Japan, was investigated by X-ray powder diffraction (XRD) [6]. It is also important to note that X-ray photoelectron spectroscopy (XPS) has been widely employed to determine the surface composition and structure of sulfur in aerosols collected at Salt Lake city [7], Jinan city of China [8], and four towns in southern Poland [9]. Although XPS provides insight into the elemental composition, empirical formula, chemical state, and electronic state of sulfur on surface aerosol, this quantitative spectroscopic technique requires not only ultra high vacuum (UHV) condition but also a considerable amount of aerosol loading on the filter. Furthermore, it is not practical to use XRD to measure the chemical state of sulfur in amorphous or poorly crystalline particle such as atmospheric aerosol. 

Recent research has found that X-ray absorption near-edge structure (XANES) can be used to determine the oxidation state of sulfur compounds even in the case of aerosol samples, which usually exist in trace levels. This innovative approach presents the potential for elucidating the very complicated chemical forms of sulfur in the natural environment. It is well known that fluorescent mode XANES (FL-XANES) is the most suitable method applied to dilute samples where “self-absorption effect” (i.e., the fluorescence X-ray signals are attenuated by reabsorption of absorber atoms in the sample) is often considered of major importance. Unlike transmission mode XANES (TM-XANES), which requires sample having X-ray absorbing atoms at high density (>5% wt), only small amount of sample is sufficient to be detected in FL-XANES. Therefore, it seems logical to conduct sulfur speciation measurement of environmental sample in FL-XANES. However, some critical facts have been identified and need to be investigated with great caution. 

Since the fluorescence excitation spectra detected by FL-XANES are more susceptible to various extraneous factors in the beam path, it is therefore crucial to estimate the signal-to-noise (S/N) ratio of target sulfur compound measured in different types of fluorescence detectors such as Germanium detector (GeD) and Lytle detector (LyD). Although the relationship between the S/N ratio of sulfur K-edge XANES spectra and the grain size was previously investigated [10], little is known about the impact of “grain size effect” on spectra absorption in aerosol sample. In addition, the availability of using sulfur speciation as a new proxy to discriminate “typical marine sediment” and “tsunami backwash deposit” will be carefully investigated. The overall aims of this study are the following.

1. To investigate the impact of GeD and LyD on S/N ratio of sulfur K-edge XANES spectra of ammonium sulfate ((NH_4_)_2_SO_4_), calcium sulfate (CaSO_4_), aluminium sulfate (Al_2_(SO_4_)_3_), manganese sulfate (MnSO_4_), zinc sulfate (ZnSO_4_·7H_2_O), potassium thiosulfate (K_2_S_2_O_3_), chromic sulfate (Cr_2_(SO_4_)_3_·15H_2_O), cobalt sulfate (CoSO_4_·7H_2_O), nickel sulfate (NiSO_4_·6H_2_O), and copper sulfate (CuSO_4_·5H_2_O) including the self-absorption effects in FL-XANES.
2. To conduct the statistical investigation of “grain size effect” on sulfur K-edge XANES spectra sensitivity in total suspended particle (TSP) and particle with diameter less than or equal to 10 microns (PM10) collected at the urban sampling sites of Bangkok, Chiang-Mai, and Hat-Yai, Thailand.
3. To evaluate the variation of sulfur speciation in both “typical marine sediments (TMS)” and “tsunami backwash deposits (TBD)” collected at the 2004 tsunami-affected coastal areas of Andaman Sea, Thailand.

## 2. Materials and Methods

### 2.1. Sample Collection and Sampling Site Descriptions

Ambient air samples were collected over periods of 6 h (09:00–15:00) using a Graseby-Andersen high volume air sampler with TSP and PM_10_ TE-6001, operating at the flow rate of 1.4 m^3^ min^−1^. TSP and PM_10_ were collected on 47 mm Whatman quartz microfiber filters (QM/A). The filters were preheated at 800°C for 12 h prior to sampling. The exposed filters were stored in a refrigerator at about 4°C until sulfur speciation analysis to prevent the evaporation of volatile compounds. All the field sampling and filters weighing were performed in compliance with the US EPA's guideline of standard operating procedure for sampling and handling of PM_2.5_ filters. It is important to note that all filters were weighed by Mettler Toledo AB204-S Analytical Balance before sending to Synchrotron Light Research Institute (Public Organization), Thailand. All the three sampling sites are located in the city center of Bangkok, Chiang-Mai, and Hat-Yai, representing the capital, the largest cities in northern and southern regions of Thailand, respectively (Table 1). Air samples were collected above the ground level at Bai-Yok Suit Hotel Observatory Site in February 18–21, 2008, at Centara Duangtawan Hotel Observatory Site in February 25–28, 2008 and at Novotel Centara Hat-Yai Hotel Observatory Site in December 17–20, 2007 as monitoring sites for Bangkok, Chiang-Mai, and Hat-Yai in that order. It is well known that traffic emissions are considered as main sources of air pollutants in Bangkok atmosphere, whilst agricultural burnings are major air pollution problems in Chiang-Mai. Furthermore, Hat-Yai is located only 30 km at the west of the Gulf of Thailand. Thus, one can safely assume that aerosol samples used in this study may consist of traffic aerosols with the mixture of industrial exhausts, biomass burning, and sea salt aerosols in the case of Bangkok, Chiang-Mai, and Hat-Yai, respectively. 

Sediment sample collection was carried out offshore along the west coast of Phang Nga province, Thailand, which was heavily affected by the 2004 tsunami [11]. The research area covers approximately 1,000 km^2^ between Thap Lamu and Pakarang Cape, up to a water depth of 70 meters. During the research cruises in November-December 2007 with RV Chakratong Tongyai, approximately 1,500 nautical miles of hydroacoustic profiles (side scan sonar, multibeam echo sounder and shallow reflection seismics with a boomer system) were recorded at the front of Pakarang Cape. In this study, two types of coastal sediments, namely, “tsunami deposit” and “typical marine sediment” were carefully identified based on previous geophysical information [10]. The sediment samples were taken with a Van-Veen-type grab sampler. Soil samples were collected from four locations at two different sites in the Lehstenbach watershed (Fichtelgebirge, Germany). In this study, three different types of terrestrial soils, namely, Endostagnic Leptic Cambisol (alumic), Histic Stagnosol (albic, alumic), and Leptic Rheic Hemic Histosol (dystric) were used for the analysis of sulfur K-edge XANES spectra for comparison purpose. Since the soil types and sampling sites have been described in detail in previous publication [12], therefore only a short summary is provided here.

### 2.2. Sulfur K-Edge XANES Experiment

XANES of the samples and standards were measured at the beamline No.8 at the Siam Photon Laboratory [13]. Photon energy of an X-ray beam transported through the beamline was scanned by InSb(111) double crystals equipped in the monochromator. The beam size illuminating the sample was 10 mm (w) × 1 mm (h). A 10 cm long ion chamber filled with a gas mixture of N_2_ (30 mbar) and He (983 mbar) was employed to measure intensity of the X-ray beam. The ion chamber absorbed only 10% of the beam intensity for ionization of the filling gas and produced small electrical current signal proportional to the beam intensity. 

Fluorescence X-rays were emitted from the sample and collected by either a 13-channel Germanium detector (GeD) or a Lytle detector (LyD). The GeD has advantage over the LyD in discrimination of X-ray fluorescence energy. With the use of a digital window, only K_*α*_ photons from sulfur were counted. The final photon counts were averaged over all the channels. Similar to the ion chamber, the LyD produces small electrical current signal corresponding not only to fluorescence X-rays from sulfur but other elements present in the sample and the scattered photons from the primary beam. The sample chamber was flowed in with He gas to reduce X-ray absorption and scattering by air. A thin polypropylene window was required to protect the GeD from He gas, which can diffuse through thin detector seal of beryllium spoiling cryostat vacuum of the detector. A minimum air gap of 5 mm was introduced for detector clearance. The distances from the sample to detector were 7 mm and 9 mm for GeD and LyD, respectively. XANES spectra were recorded from 2450 eV to 2520 eV with the energy step of 0.2 eV and energy-calibrated using the maximum absorption of iron sulfate at 2481.4 eV [14]. In addition the resolving power (E/DE) is 10,000. Absorbance was calculated by the ratio of photon counts from the fluorescent detector to the ion chamber.

For sensitivity test (NH_4_)_2_SO_4_, CaSO_4_, Al_2_(SO_4_)_3_, MnSO_4_, ZnSO_4_·7H_2_O, K_2_S_2_O_3_, Cr_2_(SO_4_)_3_·15H_2_O, CoSO_4_·7H_2_O, NiSO_4_·6H_2_O, and CuSO_4_·5H_2_O were diluted by physically mixing with boron nitride (BN) with the mixing ratios (standard: BN in percentage) of 0.1%, 1.0%, 10.0%, and 100%. The dilute standards were applied on adhesive side of the polyimide tape and placed at 45°C to the beam path in the sample chamber. Without dilution, similar approach was used for the preparation of environmental samples. The data processing and quantitative XANES analyses were conducted by ATHENA program in the IFEFFIT computer package [15]. In this study, SNR (signal-to-noise ratio) was employed to characterize the sensitivity of S-K-edge XANES spectra. “Noise” was calculated by using the standard deviation of peak intensities of preedge region (i.e., 2,450–2,475 eV), whilst the definition of “signal” was termed as the difference between main peak at 2,482 eV and the average peak intensity of preedge region (i.e., baseline). 

### 2.3. Determination of Water Soluble Ionic Species (WSIS) in Aerosol Samples

All filter samples were stored in a refrigerator at about 4°C as soon as possible after sampling was completed. This was necessary to prevent any negative artifacts caused by losses of semivolatile compounds. Also field blank filters were collected to subtract the positive artifacts due to adsorption of gas phase organic compounds onto the filter during and/or after sampling. The analysis of water soluble ionic species (WSIS) including cation (Na^+^, NH_4_^+^, K^+^, Ca^2+^) and anion (Cl^−^, NO_3_^−^, SO_4_^2−^) was performed by using Metrohm Ion Chromatography (IC) analyzer with the combination of IC 818 pump, IC 819 detector, IC 830 advance interface, and IC 833 Advanced IC Liquid Handling Pump Unit—two-channel peristaltic pump for use with the Metrohm Suppressor Module (MSM). 

Metrosep A Supp 16–250 and Metrosep C 4–250 columns were employed for the analysis of anion and cation species, respectively. Filters were cut, placed in a polyethylene vial (50 mL), and extracted with 10 mL of Milli-Q water for 30 min. The extract was filtered through 0.2 *μ*m pore size Millipore Teflon filters for cleanup. In addition, standard solutions were purchased from Fuka (Multielement Ion chromatography: number 89886.0050 and No.89316.0050 and Ammonium ion standard solution: number 09685.0250).

## 3. Results and Discussion

Ten sulfur compound standards, six aerosols, ten marine sediments, and five urban soils were analyzed by XANES in fluorescence mode detected with two different detectors. Generally, XANES can be measured in three different ways, namely, transmission, fluorescence, and electron yield modes. These three modes are widely adopted depending upon purposes. For instances, the scanning transmission X-ray microscope can be applied for imaging various biological as well as polymer samples at 55 nm Rayleigh resolution, whilst the fluorescent mode can be used for measuring X-ray absorption spectra of crystalline (*c*)-Si–C–N thin film [16, 17]. 

In the case of conventional transmission mode, the intensity of the monochromatic X-ray incident is detected before (I_0_) and after processing through the target sample (I_n_) employing ion chamber gas detectors. As a consequence, the XANES spectrum will then demonstrate the oscillation of log (I_0_/I_n_) versus energy (eV). Whilst it is widely accepted that the transmission mode is suitable for those “high density thin layer” type sample, the electron yield mode coupled with total external reflection fluorescence is generally considered as the most appropriate detector for analyzing those “high density high layer” type sample. It is also important to stress that the fluorescence detector has the greatest advantage to apply with those dilute, relatively low K-edge energies, and small samples [16]. Furthermore, a recent study reveals that the combination of high-purity Germanium (HPGe) detector and fluorescence mode can combat various analytical difficulties such as the complex signal processing electronics and limitation of sample size and amount [17]. Thus, it seems logical to apply fluorescence mode for overcoming the problems of limited sample loading with typical high-matrix containing such as environmental samples. 

### 3.1. Comparison of Sensitivity Test between GeD and LyD

**Figure 1:**
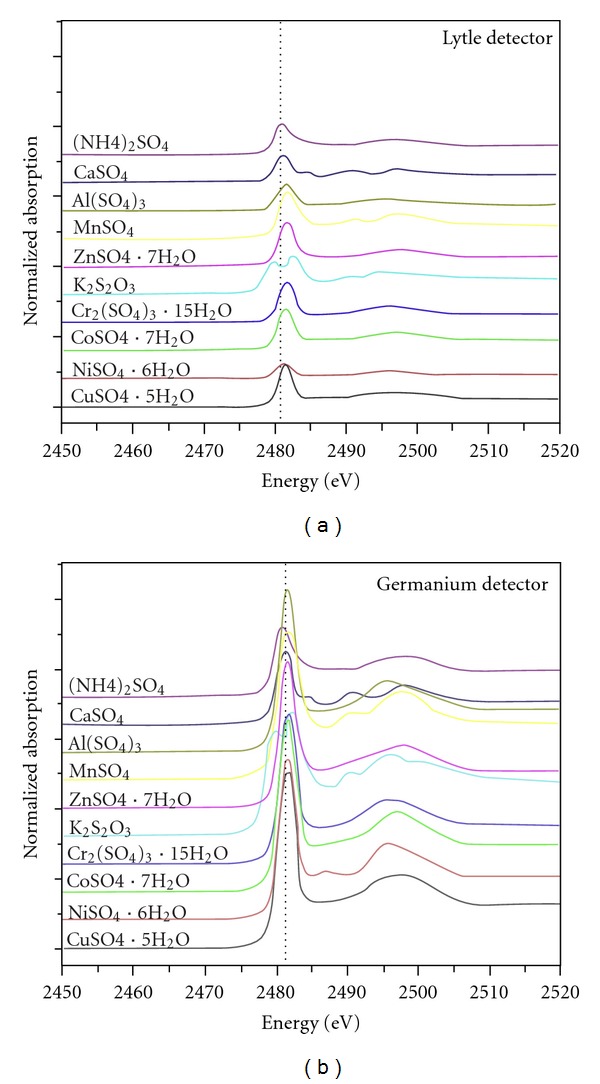
Normalized XANES spectra at sulfur K-edge spectra for (NH_4_)_2_SO_4_, CaSO_4_, Al(SO_4_)_3_, MnSO_4_, ZnSO_4_∗7H_2_O, K_2_S_2_O_3_, Cr_2_(SO_4_)_3_∗15H_2_O, CoSO_4_∗7H_2_O, NiSO_4_∗6H_2_O, and CuSO_4_∗5H_2_O measured by Germanium and Lytle detectors.

The sulfur K-edge XANES spectra for (NH_4_)_2_SO_4_, CaSO_4_, Al_2_(SO_4_)_3_, MnSO_4_, ZnSO_4_·7H_2_O, K_2_S_2_O_3_, Cr_2_(SO_4_)_3_·15H_2_O, CoSO_4_·7H_2_O, NiSO_4_·6H_2_O, and CuSO_4_·5H_2_O exhibited the main peak at 2,482 eV for both detectors. The degree of reproducibility of main peak was relatively stable within ±0.3 eV for various sulfate compounds and also consistent with other previous studies of sulfur speciation [18, 19]. For this reason it appears difficult to utilize the energy level to characterize each sulfate compounds. However, there were some differences among sulfur K-edge XANES spectra observed in the postedge region between 2,482 and 2,500 eV as displayed in Figure 1. It is likely that the change in subsequent structure must be ascribed to electron transitions with a d-type shape resonance, which is dependent on cation type associated with sulfate ions [20, 21]. The obvious difference in the sulfur K-edge XANES spectrum between K_2_S_2_O_3_ and other sulfate compounds might reflect the level of oxidation state dissimilarity. 

A crucial caution for fluorescence measurements is the selection of detector types to use in the ion chamber. In general, it is desirable to adopt LyD for measuring samples with relatively high ion count rate due to its much larger solid angle of acceptance in comparison with those of GeD. On the contrary, it is preferable to use GeD for detecting more dilute elements in samples as a consequence of recent improvement in crystallographic properties, enhancement in purity concentrations with larger axial uniformity, and enlargement in detector size [22, 23]. Additionally, the electron yield mode is often used for bulk and surface-sensitive analysis [24]. In practice, however, there are many factors that may undermine the sensitivity of XANES spectrum measured by GeD. Several authors have raised concerns on significant damping of XANES spectra of standard samples caused by self-absorption effects [25, 26]. In order to minimize this effect, it is often necessary to blend sample with an inert matrix such as BN to increase the S/N ratio. Generally, XANES spectra of ten sulfur compounds indicated that better sensitivity could be achieved in both detectors with mixing ratios of standard to BN at 0.1% (Table 2). Whilst the two detectors generated similar sensitivity for XANES spectra of (NH_4_)_2_SO_4_, CaSO_4_, Al_2_(SO_4_)_3_, MnSO_4_, ZnSO_4_·7H_2_O, Cr_2_(SO_4_)_3_·15H_2_O, CoSO_4_·7H_2_O, NiSO_4_·6H_2_O, and CuSO_4_·5H_2_O, the K_2_S_2_O_3_ was significantly lower than others with all mixing ratios of standard to BN (*P* < 0.05). 

### 3.2. Particle Size Effect on Sulfur K-Edge XANES Spectra Sensitivity in Urban Aerosol

**Figure 2:**
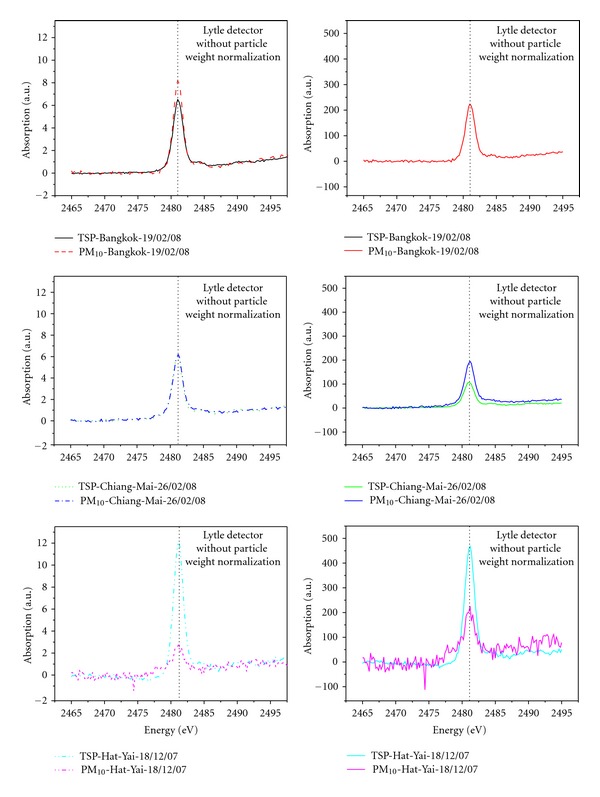
Comparison of sulfur K-edge XANES spectrum of urban aerosol collected at Bangkok, Chiang-Mai, and Hat-Yai measured by Lytle detector with and without particle weight normalization.

Recent studies have shown that the analysis of chemical speciation in aerosol can be conducted by using XANES technique [27–30]. However, only four studies have been examining sulfur K-edge XANES spectra in aerosol [31–33]. As shown in Figure 2, sulfur K-edge XANES spectra are obtained for both TSP and PM_10_ samples collected at Bangkok, Chiang-Mai, and Hat-Yai. All XANES spectra exhibited very strong symmetric peaks around 2,482 eV and shoulders nearby 2,485 eV. Generally, the aerosols examined produced less signals in the TSP mode compared with the PM_10_ mode. This can be explained by the signal damping caused by self-absorption effect of large particle as mentioned previously in Section 3.1. This effect seems to have become less pronounced with decreasing particle size and thus enhancing the signal of PM_10_ in comparison with those of TSP. It is also worth to note that differences in sensitivity between the two aerosol modes became more distinct after normalizing the signal intensity with particle weight (see Figure 2). However, there was one exception to this finding. While PM_10_ mode yielded excellent sensitivity for Bangkok and Chiang-Mai aerosols, TSP mode provided a better signal intensity than PM_10_ mode for Hat-Yai samples. Although the reason for the “sensitivity conversion” is not readily apparent, there are several possibilities behind the issue. 

Analytical results showed that the highest ionic concentrations in Bangkok aerosols were SO_4_^2−^ > NO_3_^−^ > K^+^ > Ca^2+^ and SO_4_^2−^ > Ca^2+^ > NO_3_^−^ > K^+^ for PM_10_ and TSP, respectively, whereas those of Chiang-Mai particles were following the decreasing order of SO_4_^2−^ > NO_3_^−^ > NH_4_^+^ > K^+^ and Ca^2+^ > SO_4_^2−^ > NO_3_^−^ > K^+^ for PM_10_ and TSP in that order. Previous studies found that the coarse fraction of SO_4_^2−^ is generally in the form of gypsum (CaSO_4_) reflecting the relatively high contribution of terrestrial dusts observed in Chiang-Mai TSP [34, 35]. More interestingly, sulfate was discovered as the most dominant ionic species in both types of aerosols. Since Bai-Yok Suite Hotel (i.e., Bangkok sampling site) is located in the heart of business area in Bangkok and therefore encompassed by main arteries for traffic into and out of the city center on a daily basis, it seems logical to ascribe the comparatively great input of sulfate and nitrate to vehicle exhaust emissions. Similarly, the reasonably pronounced influence of sulfate and nitrate in PM_10_ measured at Chiang-Mai was observed and thus emphasized the possibility that traffic emissions were plausibly main sources of airborne pollutants in fine particles of this city.

On the contrary, the highest contributions of Hat-Yai ionic species in TSP came from Cl^−^ > SO_4_^2−^ > Na^+^ > Ca^2+^, while those in PM_10_ were following the decreasing sequence of SO_4_^2−^ > Cl^−^ > Na^+^ > NO_3_^−^. Since Hat-Yai city is located only 30 km in the west side of coastline, it appears reasonable to presuppose the moderately high loads of Na^+^ and Cl^−^ observed in Hat-Yai aerosols as a result of sea salt aerosol production occurring above the Gulf of Thailand. The finding is comparable with prior evidence that SO_4_^2−^ steadily appears to account for 36% (PM_10_) and 24% (TSP) by mass of the total WSIS [36]. Over the past three decades, several studies suggest that SO_4_^2−^ maritime aerosols in the nucleation mode can be principally generated by *gas-to-particle conversion* (GPC), which are triggered by photooxidation of sulfur containing precursor gaseous species such as SO_2_, H_2_S, CS_2_, COS, CH_3_SCH_3_, and CH_3_SSCH_3_ [37, 38]. The highest mass percentage of particulate SO_4_^2−^ was detected in Hat-Yai TSP with the value of 22.8%, which exceeded those of Bangkok (10.6%) and Chiang-Mai (5.0%) for 2.2 and 4.6 times, respectively. Similarly, the highest mass percentage of SO_4_^2−^ in fine aerosol was observed in Hat-Yai PM_10_ with the value of 18.4%, which surpassed those of Bangkok (7.2%) and Chiang-Mai (2.8%) for 2.6 and 6.6 times correspondingly. 

These results were in good agreement with the maximum absorption of Hat-Yai samples in sulfur K-edge XANES spectra detected at 2,482 eV for both TSP and PM_10_, highlighting the positive correlation between percentage contribution of particulate SO_4_^2−^ and XANES absorption. However, it is important to stress that the ratios of SO_4_^2−^-PM_10_/ SO_4_^2−^-TSP showed the reverse sequence against those of SO_4_^2−^ mass percentages. Interestingly, the ratios of SO_4_^2−^-PM_10_/SO_4_^2−^-TSP were following the decreasing order of Chiang-Mai > Bangkok > Hat-Yai, suggesting that Hat-Yai aerosols were overwhelmed with contribution of SO_4_^2−^ in TSP mode rather than PM_10_ mode. Hence, it seems reasonable to ascribe the “sensitivity conversion” in sulfur K-edge XANES spectra as a consequence of sulfate enrichment found in Hat-Yai TSP. 

### 3.3. Variations of Sulfur K-Edge XANES Spectra in Different Soil and Sediment Types

The great Indian Ocean tsunami of December 26, 2004 was responsible for an estimated 250,000 of deaths and destroyed the coastal regions of the Indian Ocean nations. The December 26 tsunami is widely considered as the worst natural disaster to ever strike Thailand and thus has already provided traumatic impacts not only to the victims, but also to the scientists, policy makers, government officers, and several national and international organizations. Despite equitably large number of publications associated with several impacts of 2004 Indian Ocean tsunami on shore, little is known about the geochemical information of tsunami backwash deposits off shore [39–42]. Since the chemical composition records of tsunami deposits are greatly significant for the studies of both modern and past tsunami episodes, it should be of great interest to discriminate between the “typical marine sediment (TMS)” and “tsunami backwash deposit (TBD).” 

**Figure 3:**
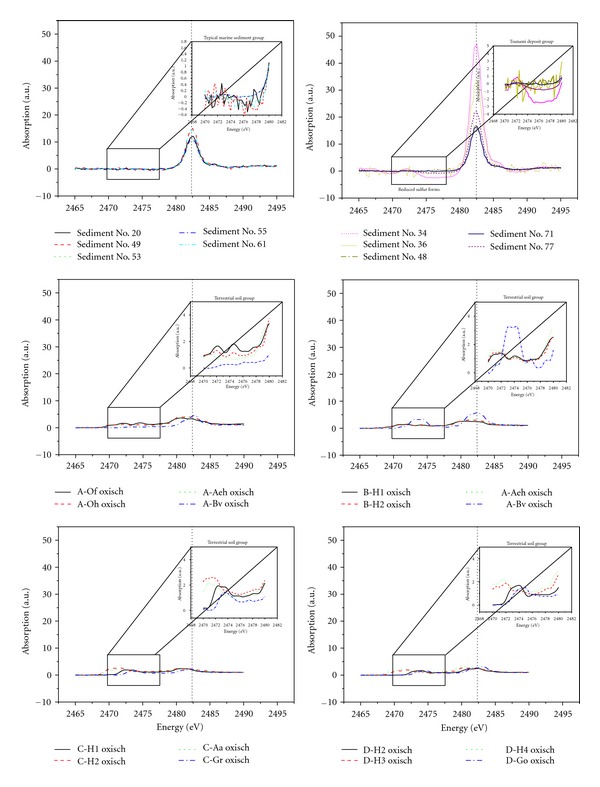
Sulfur K-edge XANES spectrum of typical marine sediment (TMS), tsunami backwash deposit (TBD), and German terrestrial soil (GS).

It was reported that the sulfur K-edge XANES spectra can be used as a “fingerprint” to characterize different types of soils [43, 44]. Thus it seems reasonable to assume that the sulfur K-edge XANES spectra can be used as a proxy to distinguish TBD from TMS. To test this hypothesis, five TMS and five TBD coupled with 16 German terrestrial soils were analyzed by the sulfur K-edge XANES in fluorescence mode as illustrated in Figure 5. The sulfur K-edge XANES spectra recorded from TMS and TBD in the present study generally showed the presence of several strong white lines at the energy level of 2,482 eV, indicating the existence of electronic oxidation state of +6, which included inorganic and ester sulfates in marine sediments. The comparatively high involvement of sulfate species in sediment is in good agreement with those reported by previous studies in Jellyfish Lake, Palau, and Oman Margin sediments [45, 46]. Interestingly, the 16 German terrestrial soils studied fluctuated noticeably in the relative proportions of the two major absorption bands in the energy ranges of 2,467–2,472 eV (i.e., the energy range of reduced oxidation state) and 2,479–2,483 eV (i.e., the energy range of oxidation state), signifying the occurrence of several oxidation states of S in the target terrestrial soils (Figure 3). Since oxidation state plays a major role in determining the electronic and physicochemical properties of sulfur species, as it exists in combination with a group of one or more other elements, the complicated S oxidation states emphasize the variety of sulfur speciation in terrestrial soil samples. 

**Figure 4:**
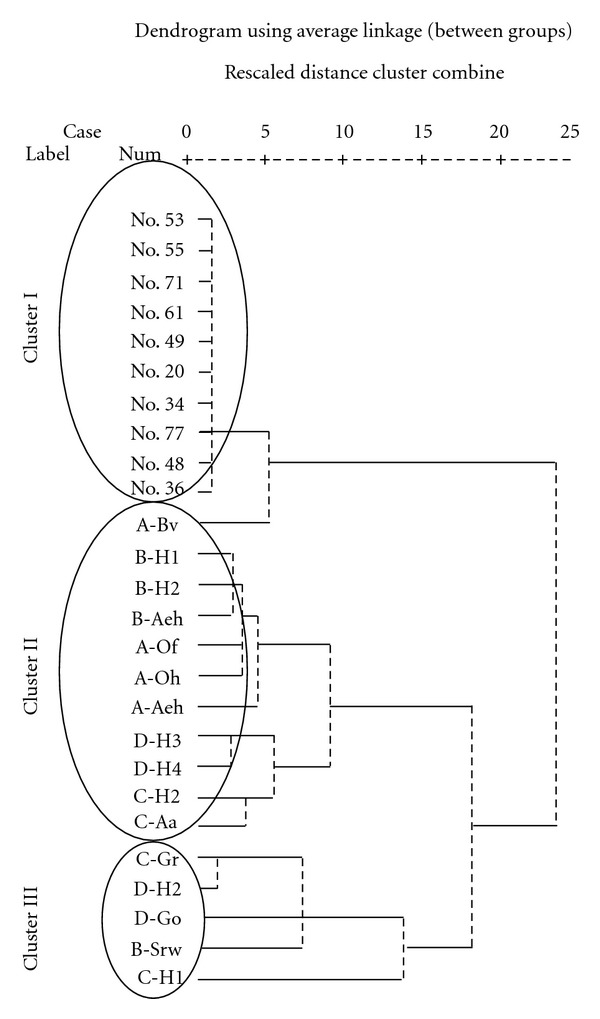
Dendrogram using average linkage (between groups) of Sulfur K-edge XANES spectrum of typical marine sediments (TMS), tsunami backwash deposits (TBD), and German terrestrial soils (GS).

With the purpose of applying the sulfur K-edge XANES spectra as an innovative “fingerprint” to discriminate TBD from TMS, hierarchical cluster analysis (HCA) was performed by using average linkage clustering. The results demonstrated in the dendrogram (Figure 4) distinguished the 26 individual sulfur K-edge XANES spectra of marine sediments and terrestrial soil samples into two major clusters. The first major cluster (i.e., Cluster I) comprised of samples No. 53, No. 55, No. 71, No. 61, No. 49, No. 20, No. 34, No. 77, No. 48, and No. 36, which can be considered as an indicative of mixing of “TBD” and “TMS.” Two major sediment types could be categorized from data similarities in line found in Cluster I. The first sediment group was characterized by samples No. 53, No. 55, No. 71, No. 61, No. 49, and No. 20 that can be considered as typical marine sediments except for sample No.71. The second one contained samples No. 34, No. 77, No. 48, and No. 36, which may be produced by the backwash of tsunami terrestrial deposits.

In order to get further insights into the sample categorization of the remaining clusters, individual dendrogram distribution of German terrestrial soils was further plotted and illustrated in Figure 4. The second major cluster (i.e., Cluster II) can be subdivided into two subclusters. The first subcluster consisted of A-Bv, B-H1, B-H2, B-Aeh, A-Of, A-Oh, A-Aeh, which were all collected at sampling site of Schlöppnerbunnen in Germany. Therefore, this subcluster represented the typical marks of Endostagnic, Leptic Cambisol (alumic), and Histic Stagnosol (albic, alumic). The second subcluster contained DH3, DH4, C-H2, C-Aa, which were found predominantly in Leptic Rheic Hemic Histosol (dystric). It was found that Cluster III was occupied by C-Gr, D-H2, D-Go, B-Srw, and C-H1, indicating the mixture of Histic Stagnosol (albic, alumic) and Leptic Rheic Hemic Histosol (dystric). Overall, HCA successfully discriminated the sulfur K-edge XANES spectra of marine sediments from those of terrestrial soils as displayed in Figure 4. 

Obviously, this dendrogram is quite useful and provides valuable information to identify the differences between marine sediments and terrestrial soils. However, this dendrogram, as well as sulfur K-edge XANES spectra, should be analyzed with great caution as oxidation process coupled with microbiological activity can alter oxidation state of S during their transport from the sampling site to the analytical laboratory. With the intention of minimizing the above-mentioned uncertainties, principal component analysis (PCA) as the multivariate analytical tool was employed to reduce a set of original variables (i.e., measured sulfur K-edge XANES spectra in sediment and soil samples) and to extract a small number of latent factors (principal components (PCs)) for analyzing relationships among the observed variables. Data submitted for the analysis were arranged in a matrix, where each column corresponds to the number of sample and each row represents the absorption intensity of sulfur K-edge XANES spectra. Data matrixes were evaluated through PCA allowing the summarized data to be further analyzed and plotted. 

**Figure 5:**
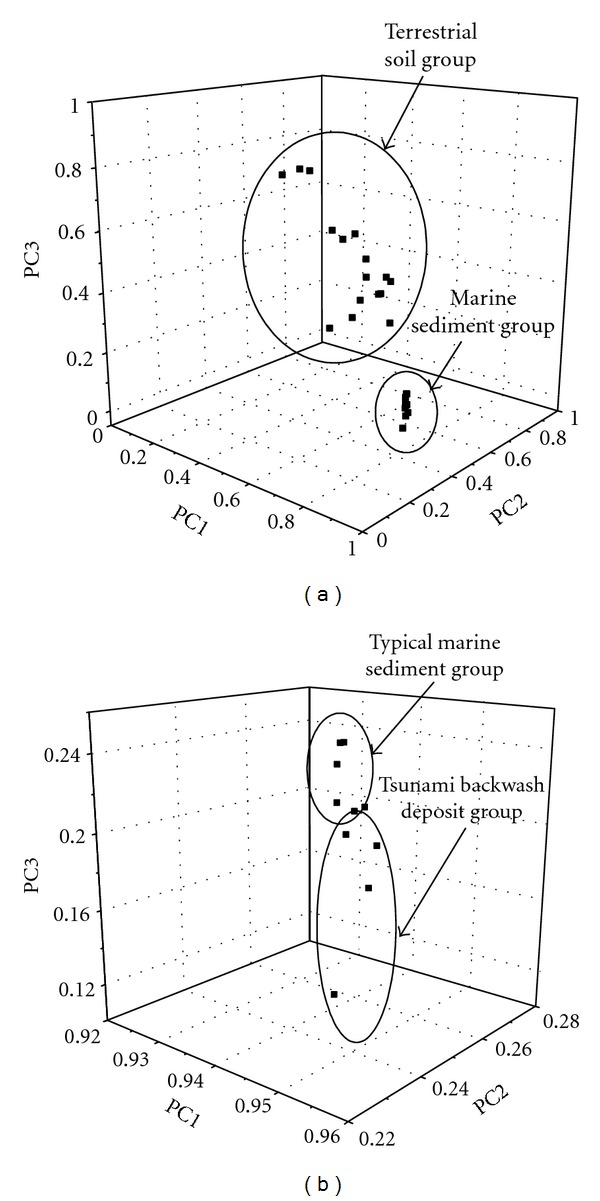
Three dimensional plots of principal component 1 (PC1), principal component 2 (PC2), and principal component 3 (PC3) of sulfur K-edge XANES spectrum of typical marine sediments (TMS), tsunami backwash deposits (TBD), and German terrestrial soils (GS).

With the aim of providing further interpretation of sulfur K-edge XANES spectra as sample fingerprints, a PCA model with three significant PCs, each representing 75.9%, 16.3%, and 4.52% of the variance, thus accounting for 96.7% of the total variation in the data, was calculated. After plotting factor scores of PC1, PC2, and PC3 in three dimensions, some distinctive features of data clustering were appeared. Firstly, 3D plots of Endostagnic, Leptic Cambisol (alumic), Histic Stagnosol (albic, alumic), and Leptic Rheic Hemic Histosol (dystric) are highly deviated from the Andaman coastal sediments in both TMS and TBD as illustrated in Figure 5(a). Secondly, a grouping of 3D plots of Andaman marine sediments can be further separated into two groups, which are (1) samples No. 20, No. 34, No. 36, No. 48, No. 49 (i.e., TMS) and (2) samples No. 53, No. 55, No. 61, No. 71, No. 77 (i.e., TBD) as displayed in Figure 5(b). As a consequence, it appears rationale to conclude that the combination of HCA and PCA could successfully distinguish German terrestrial soils from Andaman marine sediments, and thus enhancing the reliability of employing the sulfur K-edge XANES spectra as a new “fingerprint” to characterize environmental samples. 

## 4. Conclusions

Generally, XANES spectra of (NH_4_)_2_SO_4_, CaSO_4_, Al_2_(SO_4_)_3_, MnSO_4_, ZnSO_4_·7H_2_O, K_2_S_2_O_3_, Cr_2_(SO_4_)_3_·15H_2_O, CoSO_4_·7H_2_O, NiSO_4_·6H_2_O, and CuSO_4_·5H_2_O standards indicated that better sensitivity could be achieved in both detectors with mixing ratios of standard to BN at 0.1%. There were no significant differences in term of instrumental sensitivity detected by both Lytle and Germanium detectors. Further investigation was conducted to assess the sensitivities of sulfur K-edge XANES spectra as functions of atmospheric particle sizes. For urban aerosols, the S/N ratios of sulfur K-edge XANES spectra are anticorrelated with atmospheric particle sizes. However, this concept cannot be applied to sulfate-rich coarse particle-like maritime TSP. This study has proved that the sensitivities of sulfur K-edge XANES spectra were positively correlated with sulfate content in particles and negatively associated with atmospheric particle sizes. In addition, the combination of advanced statistical methods such as HCA and PCA evidently separated German terrestrial soils from Andaman marine sediment, and moderately distinguished typical marine sediments from tsunami backwash deposits.

## Figures and Tables

**Table 1:** Location and general description of sampling sites.

Sampling location	Sample number	Sample type	Sample descriptions	Latitude	Longitude
Bangkok, Thailand		PM_10_ and TSP	Traffic and industrial aerosols	13° 44′ 496′′ N	100° 59′ 819′′ E
Chiang-Mai, Thailand		PM_10_ and TSP	Traffic and biomass burning aerosols	18° 47′ 015′′ N	98° 59′ 519′′ E
Hat-Yai, Thailand		PM_10_ and TSP	Traffic and maritime aerosols	07° 00′ 087′′ N	100° 27′ 262′′ E
PSU, Thailand		PM_10_ and TSP	Urban background aerosols	07° 21′ 280′′ N	100° 29′ 533′′ E
Andaman Sea, Thailand	20	Marine sediment	Coarse silt, moderately sorted (water depth 57.80 m)	08° 49′ 296′′ N	97° 59′ 819′′ E
Andaman Sea, Thailand	34	Tsunami deposit	Coarse sand, well sorted (water depth 16.70 m)	08° 43′ 835′′ N	98° 11′ 916′′ E
Andaman Sea, Thailand	36	Tsunami deposit	Very coarse sand, well sorted (water depth 17.30 m)	08° 43′ 875′′ N	98° 10′ 945′′ E
Andaman Sea, Thailand	48	Tsunami deposit	Coarse sand, well sorted (water depth 17.80 m)	08° 42′ 901′′ N	98° 09′ 988′′ E
Andaman Sea, Thailand	49	Marine sediment	Muddy fine sand, poorly sorted (water depth 62.00 m)	08° 30′ 222′′ N	97° 59′ 124′′ E
Andaman Sea, Thailand	53	Marine sediment	Medium sand, moderately sorted (water depth 59.80 m)	08° 33′ 421′′ N	97° 58′ 422′′ E
Andaman Sea, Thailand	55	Marine sediment	Muddy medium sand, poorly sorted (water depth 57.10 m)	08° 33′ 810′′ N	98° 00′ 908′′ E
Andaman Sea, Thailand	61	Marine sediment	Muddy medium sand, poorly sorted (water depth 45.60 m)	08° 35′ 863′′ N	98° 03′ 630′′ E
Andaman Sea, Thailand	71	Tsunami deposit	Coarse sand, moderately sorted (water depth 15.80 m)	08° 42′ 928′′ N	98° 10′ 906′′ E
Andaman Sea, Thailand	77	Tsunami deposit	Silty coarse sand, poorly sorted (water depth 13.90 m)	08° 42′ 569′′ N	98° 11′ 680′′ E
Schlöppnerbunnen, Germany	A-Of oxisch	Terrestrial soil	Endostagnic, Leptic Cambisol (alumic)	50° 08′ 140′′ N	11° 53′ 070′′ E
Schlöppnerbunnen, Germany	A-Oh oxisch	Terrestrial soil	Endostagnic, Leptic Cambisol (alumic)	50° 08′ 140′′ N	11° 53′ 070′′ E
Schlöppnerbunnen, Germany	A-Aeh oxisch	Terrestrial soil	Endostagnic, Leptic Cambisol (alumic)	50° 08′ 140′′ N	11° 53′ 070′′ E
Schlöppnerbunnen, Germany	A-Bv oxisch	Terrestrial soil	Endostagnic, Leptic Cambisol (alumic)	50° 08′ 140′′ N	11° 53′ 070′′ E
Schlöppnerbunnen, Germany	B-H1 oxisch	Terrestrial soil	Histic Stagnosol (albic, alumic)	50° 08′ 140′′ N	11° 53′ 070′′ E
Schlöppnerbunnen, Germany	B-H2 oxisch	Terrestrial soil	Histic Stagnosol (albic, alumic)	50° 08′ 140′′ N	11° 53′ 070′′ E
Schlöppnerbunnen, Germany	B-Aeh oxisch	Terrestrial soil	Histic Stagnosol (albic, alumic)	50° 08′ 140′′ N	11° 53′ 070′′ E
Schlöppnerbunnen, Germany	B-Srw oxisch	Terrestrial soil	Histic Stagnosol (albic, alumic)	50° 08′ 140′′ N	11° 53′ 070′′ E
Schlöppnerbunnen, Germany	C-H1 oxisch	Terrestrial soil	Leptic Rheic Hemic Histosol (dystric)	50° 08′ 140′′ N	11° 53′ 070′′ E
Schlöppnerbunnen, Germany	C-H2 oxisch	Terrestrial soil	Leptic Rheic Hemic Histosol (dystric)	50° 08′ 140′′ N	11° 53′ 070′′ E
Schlöppnerbunnen, Germany	C-Aa oxisch	Terrestrial soil	Leptic Rheic Hemic Histosol (dystric)	50° 08′ 140′′ N	11° 53′ 070′′ E
Schlöppnerbunnen, Germany	C-Gr oxisch	Terrestrial soil	Leptic Rheic Hemic Histosol (dystric)	50° 08′ 140′′ N	11° 53′070′′ E
Schlöppnerbunnen, Germany	D-H2 oxisch	Terrestrial soil	Leptic Rheic Hemic Histosol (dystric)	50° 08′ 380′′ N	11° 51′ 410′′ E
Schlöppnerbunnen, Germany	D-H3 oxisch	Terrestrial soil	Leptic Rheic Hemic Histosol (dystric)	50° 08′ 380′′ N	11° 51′ 410′′ E
Schlöppnerbunnen, Germany	D-H4 oxisch	Terrestrial soil	Leptic Rheic Hemic Histosol (dystric)	50° 08′ 380′′ N	11° 51′ 410′′ E
Schlöppnerbunnen, Germany	D-Go oxisch	Terrestrial soil	Leptic Rheic Hemic Histosol (dystric)	50° 08′ 380′′ N	11° 51′ 410′′ E

**Table 2:** Signal-to-noise ratios (S/N) of (NH_4_)_2_SO_4_, CaSO_4_, Al(SO_4_)_3_, MnSO_4_, ZnSO_4_ ∗ 7H_2_O, K_2_S_2_O_3_, Cr_2_(SO_4_)_3_ ∗ 15H_2_O, CoSO_4_ ∗ 7H_2_O, NiSO_4_ ∗ 6H_2_O, and CuSO_4_ ∗ 5H_2_O detected by GeD and LyD.

Mixing ratio % (sample : BN)	Germanium	Germanium	Germanium	Germanium	Lytle	Lytle	Lytle	Lytle
	0.1	1.0	10.0	100.0	0.1	1.0	10.0	100.0
Al_2_(SO_4_)_3_	4.296	3.837	3.241	2.642	4.529	3.935	3.440	3.020
CaSO_4_	3.719	3.801	3.213	2.984	3.969	3.731	3.424	2.951
ZnSO_4_	3.650	3.598	2.893	2.714	3.532	3.640	3.056	2.894
NiSO_4_	4.308	3.965	3.334	3.136	4.196	4.145	3.503	3.385
NH_4_SO_4_	4.711	2.745	2.509	1.581	4.553	2.560	2.643	1.923
K_2_S_2_O_3_	2.431	2.345	1.868	1.593	3.582	2.399	2.264	1.892
FeSO_4_	3.936	3.551	3.216	3.021	3.778	3.749	3.409	3.132
CuSO_4_	4.301	4.022	3.483	3.100	4.027	4.229	3.660	3.407
CrSO_4_	4.120	4.021	3.539	2.915	3.865	4.033	3.633	3.074
CoSO_4_	4.212	3.810	3.348	3.144	4.333	4.032	3.241	3.203

Average	3.968	3.569	3.064	2.683	4.036	3.645	3.227	2.888
Stdev %	15.7	16.0	16.9	22.4	9.0	17.6	14.0	18.8
